# Halitosis - an assessment protocol proposal

**DOI:** 10.1016/S1808-8694(15)31180-0

**Published:** 2015-10-19

**Authors:** Ana Cristina Coelho Dal Rio, Ester Maria Danielli Nicola, Antônio Roberto Franchi Teixeira

**Affiliations:** 1 Specialist, Dentist - CECOM-UNICAMP; 2 PhD. Professor of Otolaryngology and Head and Neck Surgery - Dept. of OPHT / ENT - FCM - UNICAMP, Coordinator of the Graduate Course - Medical Sciences - FCM-UNICAMP, Coordinator of the Laser Medicine Multidisciplinary Unit - HC-UNICAMP; 3 PhD in Surgery - FCM-UNICAMP, Adjunct Professor - Medical School of Jundiaí

**Keywords:** causes of halitosis, halitometry, halitosis, bad breath

## Abstract

Halitosis is an embarrassing symptom with a significant social impact. Halitosis affects millions of people worldwide and many resources are spent annually in products to improve halitus, unsuccessfully. The study of halitosis in a scientific basis is justified once halitosis causes social restriction, decreases life quality and may be an indication of serious diseases.

**Aim:**

To elaborate a protocol for halitosis assessment in order to minimize costs, avoid unnecessary tests and provide a guideline for diagnosis. METHODS: The protocol was created based on the literature and on the authors' personal experiences, adopting an evidence-based anamnesis.

**Results:**

There are many causes of halitosis and most of them are related to the oral cavity; others are related to otolaryngologic and respiratory diseases. Gastrointestinal diseases, liver/renal impairment and other metabolic syndromes are less frequent, but also important causes of halitosis.

**Conclusion:**

There are important costs involved in halitosis assessment and treatment, including medical appointments, specialist assessment, and complementary tests. Such costs would be minimized by adopting a protocol of evidence-based anamnesis and a flowchart for a rational clinical investigation.

## INTRODUCTION

Halitosis is a Latin word which means halitus (breathed air) and osis (pathologic alteration)^1^. One of the first recordings pertaining to halitosis is in the Bible where Job (19:17) regretted: “My bad breath is unbearable to my wife…” The philosopher Plutarch, in his book WRITTING ABOUT MORALITY, has written that Heron of Syracuse when informed about his bad breath by his doctor turned to his wife and said: “Why have not you told me before that my bad breath hurts you every time that I kiss you?” In addition, her answer was “I've always thought that all men had this horrible odor”^2^. Almost two thousands years ago the Jewish could cancel the wedding contract (ketuba) if they realized that the woman had bad breath. The Islamic theology emphasizes the importance of the siwak (a special device for cleaning the mouth), recommending its use during the Ramadan starving period in order to prevent halitosis^3^.

Halitosis is an unpleasant alteration of the halitus for the person who has the symptom and for related people either, being a pathological condition or not 4. It is also known as fetid halitus, stinking mouth, bad breath or oral malodor^2^,^5^. Halitosis is a common complaint among adults of both genders all over the world. It has a multifactorial etiology, but its main cause is the decomposition of the organic material by microorganisms of the oral cavity^6^. One of the pioneers in halitosis research was Howe who described this symptom in 1874 and since then, halitosis has been considered a clinical entity4. The majority of the citations concerning to halitosis before 1930 were not confirmed by facts or studies, however were perpetuated by literature^7^. In 1934, Fair and Wells created an instrument called osmoscope, which was used for measurements of odor density in a subjective and semi quantitative way^7^. During the 40's and 50's, Fosdick and his associates from Northeastern University, used the osmoscope to conduct numerous studies and produced valuable information about halitosis causes and conditions that could be related^7^. These authors concluded that, although halitosis can have physiologic and/or pathologic systemic causes, the main cause of halitosis is physiologic and has to do with the oral cavity^7^,^8^. Joe Tonzetich did the first studies in the 60's searching for halitosis causes^9^. This author also described some clinical features related to bad breath and in late 70's he initiated studies about the Volatile Sulphur Compounds (VSCs)^8^.

During the 60's and 70's the researches concentrated, mainly, on chemical and instrumental analysis in an attempt to identify the basic halitosis's compounds. By this time, a highly sensitive and specific chromatographic gas method was adapted for direct measurements of the volatile sulphur compounds in the saliva and breath^7^. Initial studies with the saliva were developed. Adding substrate inhibitors and enzymes, it has been determined that the VSCs were the main substances responsible for the malodor of putrefied saliva^9^. In the 1970's, the gas chromatography was the most sensitive instrument used in clinical research. The gas chromatography made possible to identify and measure directly the individual VSCs components (H2S- hydrogen sulfide, CH3SH -methanethiol and CH3SCH3- dimethylsulfide) in the halitus^7^.

**Figure 1:**
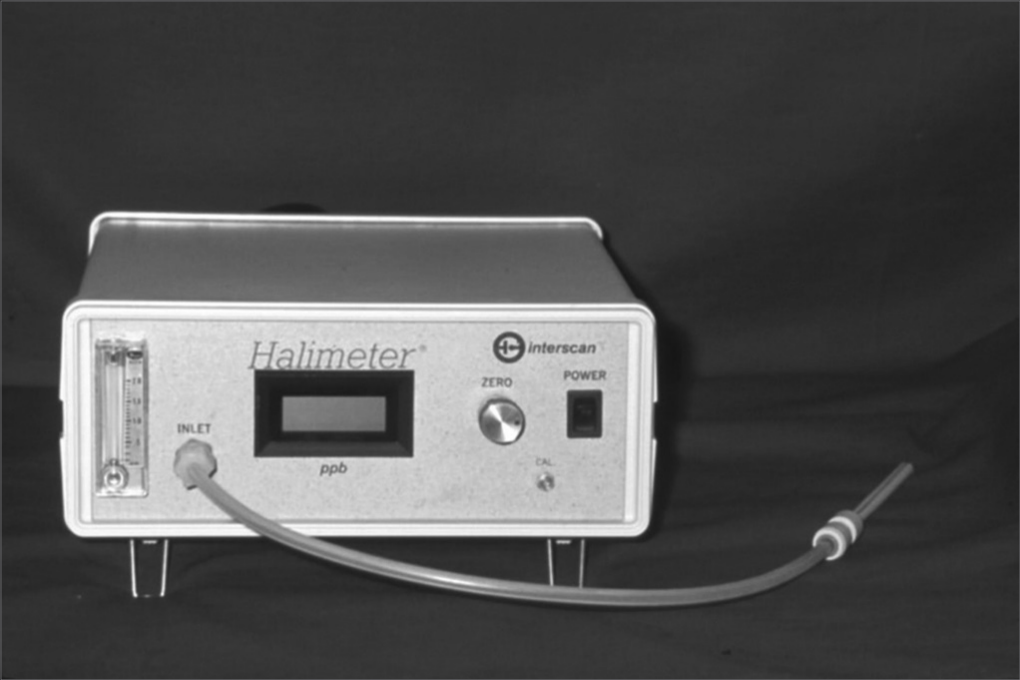
Halimeter: an objective measurement of Volatile Sulphur Compounds (VSC)

The organoleptic assessment is a subjective measurement. It is a very good qualitative method, however not very precise concerning to quantity. It depends on the examiner's olfaction accuracy what may change in case of influenza, environment humidity, etc^10^. The patient is asked to come to the office during the period he/she feels the halitus is at its worst. He is also asked not to use mouth rinses and the toothbrush at least two hours before the test. The test consists on asking the patient to breathe deeply inspiring the air by nostrils and expiring by mouth, while the examiner sniffs the odor at a distance of 20 cm, considering it unpleasant or not in a scale of 0 to 510. Self-examination can be relevant as it involves the patient in the process. Licking his/her own wrist, smelling it after a while, reflects the saliva contribution to oral malodor. Because organoleptic assessment is a subjective measurement, examiners must use an objective test such as Halimeter® (Figure 1) or BANAÒ test to confirm results10. The technology development represented a considerable advance concerning to halitosis diagnosis and assessment of the treatment used.

**Figure 2:**
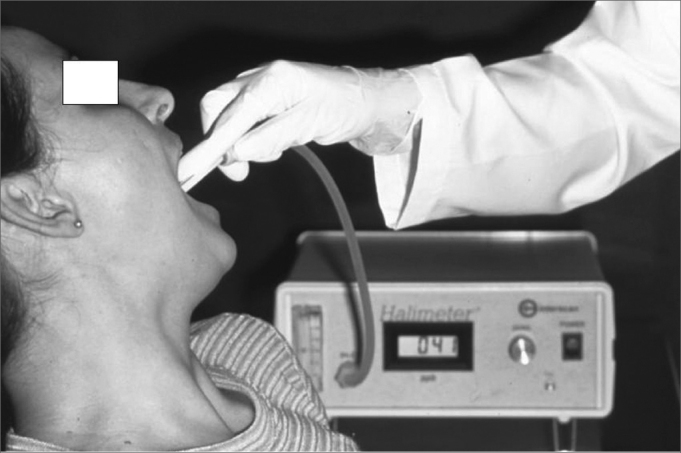
Halitometry Technique - Illustration of the procedure to measure VSC with the Halimeter device.

Devices for objective assessment of oral malodor have been developed. They can be very useful to detect the VSC quantitatively and correlate them with specific diseases, their impact over the quality and intensity of oral malodor^4^. The Halimeter® is the most common device used nowadays. It has a digital display, which records the quantity of VSC in parts per billion (ppb). Halimetry values (Figure 2) are considered normal when they are below 150ppb. The Halimeter® does not record all the odor vectors present in the breathed air, so it does not dispense a good anamnesis and clinical examination. The use of mouth rinses or toothpaste can produce a bias in the measurement by the Halimeter®^11^.

The BANAÒ test (enzymatic method benzoyl-arginine-naphtylamide) is a very practical tool to assess bacterial proliferation in the gingival sulcus and its positiveness is strongly related to periodontal diseases^12^. This test, when compared to the VSC HalimeterÒ test, can diagnose halitosis when the Halimeter test is negative. This data suggest that BANA Òcould be complementary to HalimeterÒ^13^. Tests to assess halitosis are summarized in Table 1.

**Table 1:** Most common tests used for halitosis assessment.

TEST	ADVANTAGES	PROBLEMS
ORGANOLEPTIC	good in subjective assessment	not precise in quantity; depends on the examiner's olfaction accuracy
VSC HALIMETERÒ	good in quantifying values	does not record all the odor vectors present in the breathed air
BANAâ TEST	complementary test to halimetry	needs a complementary assessment

## MAIN CAUSES OF HALITOSIS

### Oral Diseases Related To Halitosis

Oral cavity pathologies that can cause halitosis are, among others: dental cavities, periodontal disease, tongue coating, exposed tooth pulps, extractions/healing wounds, interdental food impaction, dentures kept at night or not regularly cleaned, restorative crowns which are not well adapted, cysts with fistula draining into the mouth, oral cancer and ulcerations. Most of these factors cause halitosis due to tissue breakdown, putrefaction of amino acids and decreasing of saliva flow. All these conditions result in the release of volatile sulphur compounds (VSC)^10^,^14^.

The oral microbiota has been researched because bacteria play a very important role in halitosis. Coccus and bacilli, Gram positive and Gram negative^15^ compound the normal oral microbiota. The oral microbiota associated with halitosis is predominantly anaerobic Gram negative, because their final metabolism products are the volatile sulphur compounds (VSC)^6^.

Tongue coating is made of concentrated saliva, bacteria, exfoliated epithelium cells, food debris and remains over the tongue dorsum^4^,^10^. Tongue coating is also responsible for the oral malodor and anatomical variations of the tongue as fissured tongue, hairy tongue, and ulcerated tongue can contribute to worsen oral malodor^10^. Nowadays, tongue scrappers are a very common device used by people in order to clean the tongue and avoid coating formation and consequently decrease halitosis4. The inadequate use of scrappers can lead to tongue ulceration, that can make halitosis worse and can lead to a very uncomfortable sensation when eating acidic or bitter food.

### Otolaryngology and Respiratory Diseases Related to Halitosis

Halitosis is a very common complaint among ENT patients. The main causes of halitosis related to the oronasal cavity are acute viral or bacterial pharyngitis, chronic/purulent tonsillitis, retropharyngeal abscesses, deep crypts of the tonsils, caseous retention, chronic/purulent sinusitis, post-nasal drip, foreign body in nasal or sinusal cavity and ozena. These pathologies cause halitosis mainly due to bacterial action, which lead to putrefaction of the tissues and production of volatile sulphur compounds (VSC)^4^,^10^. Fetid samples of the tongue dorsum coating were compared with nasal mucus and showed the same composition3. Nasal obstruction leads to mouth breathing causing dryness of the mouth. A dry mouth causes more epithelium cells exfoliation, xerostomia, tongue coating and therefore increases the production of volatile sulphur compounds (VSC)^10^.

The deep crypts of the tonsils have to do with caseous retention. Chronic caseous tonsillitis is a pathology, which symptoms are described by patients as a foreign body in the throat, throat irritation, and halitosis complaint is very high^16^,^17^. Recently, the modified cryptolysis technique with CO_2_ laser as a conservative method for the treatment of chronic caseous tonsillitis has been proved a very safe and effective method, preserving the tonsilar parenchyma and decreasing caseous retention^16^.

Concerning to bronchi and lungs, there are some pathologies such as chronic bronchitis, bronchial carcinoma, bronchiectasis that cause tissue necrosis and ulcerations, producing malodorous gases, which are expired causing halitosis^3^,^8^. In addition, objects aspirated accidentally can lead to lung abscess formation and consequently produce halitosis bodies^10^.

### Digestive Diseases Related to Halitosis

Many digestive diseases are traditionally associated with halitosis. Reflux esophagitis, hiatal hernia, Zencker diverticulum, achalasia are associated. Actually, steatorrhea or other malabsorption syndromes, which cause excessive flatulence, are the most important causes of halitosis concerning gastrointestinal diseases^4^,^10^. Specialists and internists often require gastroenterological assessment when facing a halitosis complaint. Endoscopy is one of the most widely requested tools in halitosis investigation^18^.

Endoscopy is important to assess gastroesophageal reflux disease (GERD) and hiatal hernia, gastritis, duodenitis, ulcers, carcinomas and helicobacter infection^19^.

In GERD, an improper function of the gastroesophageal inferior sphincter allows acid and non-acid stomach contents to flow back into the esophagus. This alteration could result in esophageal mucosal break down. These areas can be inhabited by bacteria, causing the production of volatile sulphur compounds. In some cases, esophagus sphincter pathologies can cause halitosis due to putrefaction of the trapped food debris and food stasis^10^,^19^,^20^.

The Helicobacter pylori infection has been associated with breath malodor; however, it is still controversial^18^,^19^,^21^,^22^. Some studies correlate H. pylori infection and altered VSC halitometries. There is some evidence that halitosis complaint in H. pylori-positive non-ulcer dyspepsia could mean H. pylori eradication^18^. Nevertheless, H. pylori has a high urease activity, which explains the pH increase and the lowered solubility of many malodorants^19^. This fact does not prove that H. pylori causes halitosis by itself. Indeed some authors believe that there is no convincing evidence that oral malodor can be linked to H. pylori infection^21^.

The presence of clots or bleeding points at any part of the digestive system can cause halitosis due to the deterioration of blood^10^. Therefore, any causes of gastrointestinal bleeding (tumors, inflammatory diseases, parasites) can cause halitosis.

Liver cirrhosis is characterized by the irreversible damage of the liver parenchyma resulting in the accumulation of ammonia. Ammonia reaches lungs through expired air, causing characteristic halitosis^8^. Generally, patients in hepatic encephalopathy have a characteristic breath scent.

The main causes of halitosis are summarized in Table 2.

**Table 2:** This table summarizes the distinct causes of halitosis.

LOCALIZATION	FREQUENCY	DISEASES
MOUTH	90%	Dental caries, periodontal diseases, tongue coating, exposed tooth pulps, healing wounds, interdental food impaction, dentures not cleaned properly, restorative crowns not well adapted, ulcerations, fistula, and oral cancer.
ENT AND RESPIRATORY SYSTEM	8%	Pharyngitis, tonsillitis, sinusitis, foreign body in nasal or sinusal cavity, bronchitis, bronchial carcinoma, bronchiectasis.
DIGESTIVE SYSTEM	1%	Regurgitation esophagitis, hiatal hernia, helicobacter pylori infection.
OTHERS	1%	Kidney insufficiency, halitophobia, trimethylaminuria, diabetes.

## OTHER CAUSES

Renal impairment is normally a result of a chronic glomerulonephritis, which damage the glomerular function, leading to an increased urea level in the blood. Breathed air is described as ammonium-like breath and generally is accompanied by complaints of dysgeusia (salty taste)^10^.

Diabetes can result in accumulation of ketone bodies, which are breathed out producing a very characteristic halitus, moreover, diabetes causes dry mouth. In addition, diabetes and other insulin-resistance states are related to impaired secretion of body fluids, like tear and saliva. There is a decrease in saliva production and xerostomia can occur^23^.

Trimethylaminuria or “fish odor syndrome” is a genetic metabolic disorder characterized by a failure in the oxidation route from trimethylamine (TMA) to trimethylamine N-oxide (TMA-O) in the liver. This occurs due to a mutation in the FMO^3^ gene. High levels of TMA in urine and others body fluids confer that typical unpleasant, intermittent characteristic fishy odor to the breath^4^,^10^.

Tumor lesions in any part of the body also produce volatile gases due to the necrosis process. These gases are expired in the breathed air causing halitosis and that is the reason why halitosis can indicate the presence of serious diseases^4^,^10^,^24^.

## SOCIAL IMPACT

The social impact of halitosis is one of the reasons for so much research. It is very embarrassing for the patients, making them feel insecure to relate to other people and decreases their life quality. It is also embarrassing for relatives and friends of people who have halitosis. In addition, the presence of halitosis can indicate the existence of other pathologies that must be diagnosed and treated as soon as possible^24^. It has been estimated that more then 85 million people suffer with halitosis. People spend over 2 billion dollars per year buying products to mask halitosis^25^. Such costs would be minimized in adopting an anamnesis based on evidence and a rational flowchart of clinical investigation. The vast majority of patients first look for help in traditional medicine, chewing gum and non-medical advice, which are not successful strategies. The evidences show very poor results using these strategies^3^. Many patients ask for guidance when consulting a general physician, a gastroenterologist and an ENT specialist. It is very important for these professionals to have a rational approach in halitosis investigation, because the causes are many, the patients are usually frustrated and good results mainly depend on attacking the origin of the problem.

## PROPOSAL FOR A RATIONAL PROTOCOL

The intention of this protocol is to assess the main causes of halitosis concerning their frequency and importance. A logical knowledge organization must be kept to avoid diagnosis failures and useless/expensive tests.

Initially a physician must have in mind that halitosis complaint is very common in the general population. Nevertheless, there is a bias concerning differences between true halitosis and “bad taste in the mouth”. Some patients look for halitosis treatment due to relatives/friends warnings and others due to self-awareness. It has to be considered the level of confidence in the information given by relatives/friends. In our experience, some cases have been mistreated due to biased information given by an unhappy consort. Moreover, there is physiologic halitosis,4 which is sometimes misinterpreted as a disease, and it can be normal. The halitosis most people experience when wake up is considered physiologic once it disappears after eating and/or brushing teeth. It is considered physiologic because of the decreased salivary flow, the increased putrefaction process at night and because of the long period of starvation while sleeping. If it persists even after eating or brushing teeth, further investigation is necessary^24^.

People complaining of halitosis sometimes do not have it. Fictitious halitosis, also known as halitophobia is an imaginary halitosis^10^. These patients when undergo physical examination, organoleptic /VSC halimeter tests often have normal results. It is more a psychological problem, so called Olfactory Reference Syndrome^26^. The Olfactory Reference Syndrome is a complex psychological disease related to alteration of corporeal consciousness that leads to social isolation and needs specific treatment^26^. These patients must be referred to psychological assessment. There is no point for further investigations because no organic or pharmacological treatment will be effective since this is a psychological symptom.

Because of this, it is mandatory to perform a very good anamnesis/history and an objective test in all patients with halitosis complaint. In our experience VSC halitometry is most used, because it gives a numerical index.

The oral cavity is responsible for 90% of the cases of halitosis, the respiratory tract is responsible for 8% and the gastrointestinal tract and others are responsible for only 2%^4^,^8^,^10^. Since the mouth is the main source of halitosis, a specialized oral/ nasal investigation is essential. Unfortunately, not any dentist is capable to make a complete assessment concerning halitosis. Mild affections as gingivitis, bacterial plaques and tongue coating diseases may not be detected in a not focused dental assessment. Recently a new field of specialization in Halitosis in Dentistry Schools has been introduced.

The otholaryngologist is helpful in halitosis assessment and treatment. Once more, when the professional is not focused in the disease some important information may be lost. Chronic sinusitis and tonsillitis are very common sources of halitosis. New approaches like computed tomography (CT) scans might be useful in detecting sinusitis not seen in a normal sinus X-ray. Chronic caseous tonsillitis (CCT) is very much related to halitosis^16^,^17^. Tonsillectomy, although being a very common procedure in ENT surgical routine, still offer risks such as bleedings and complications with anesthesia^29^. New conservative approaches as laser ablation can restore normal halitus, avoiding tonsillectomy^16^,^17^.

Nowadays, because of all the problems related to modern life styles, people do not drink and eat properly. The majority of people who complain of halitosis do not drink enough water or do not drink at all, replacing it for beverages or soft drinks. It causes an impairment of saliva flow due to water scarcity. They also eat too fast, not chewing adequately. In addition, work routines and schedules do not facilitate oral hygiene. Lots of working people do not have a specific time to eat properly, and they fast for long periods. Besides that, habits such as smoking and alcoholic ingestion make things even worse. It is called specific halitosis when there is a strict relationship with a substance smoked or ingested^27^. These are examples of substances that can cause halitosis: tobacco, cigars, pipes, alcohol, and marijuana. Some of these substances can cause xerostomia as a side effect, marijuana, for instance^28^. Chain smokers, mainly cigarettes smokers, can have their tongue transformed into hairy tongue due to papilla atrophy. This may retain more food particles and epithelium cells debris, enhanced by xerostomia provoked by smoking^28^. Alcoholic beverages cause specific halitosis because they produce volatile compounds after being metabolized. Normally this kind of halitosis occurs in a couple of minutes or hours after the beverage is ingested. Alcohol also can alter the intestinal flora, producing halitosis due to fermentation^27^. Other examples of food that can cause specific halitosis are garlic and onion in an excessive intake.

When facing true halitosis a diet evaluation is essential. A nutritionist assessment is essential and there is no virtual chance of cure for these patients without alimentary re-education.

After that, if halitosis persists, an internist must be assessed. Endoscopy is one of the most widely examinations prescribed, but its efficacy has not been proved yet^19^. It is clear that in some cases of GERD and hiatal hernia, reflux can contribute to halitosis^19^. There are no formal evidences correlating Helicobacter pylori infection and halitosis and its eradication in this case is still controversial^19^,^21^,^22^. Gastroenterologists can contribute in halitosis assessment because some liver diseases can be involved. Liver cirrhosis affect protein metabolism. Animal proteins are decomposed in the liver and some metabolites (ammonia) can contribute to halitosis. Diabetes can contribute to ketosis and a typical rotten apple halitus can be detected. Uremic patients also have a typical smell, because they have amonnemia in several body fluids. Those are rare but important causes of halitosis^4^,^10^.

In a few patients, a respiratory evaluation must be necessary. Children sometimes aspirate small objects. It could lead to abscess formation and a bad breath is typically detected. Other lung affections as tuberculosis, blastomicosis and fugal abscesses also can contribute to halitosis^4^,^10^.

In our personal experience, most cases of halitosis can be detected an properly treated by an experienced dentist and by an ENT specialist. In approximately half of the patients, an otolaryngologist contribution is essential. Most patients referred to a gastroenterologist underwent useless and expensive endoscopies. Thousands of unnecessary endoscopies are performed every year in patients complaining of halitosis. Although there is a significant effort trying to correlate halitosis and H. pylori infection, there is no supporting evidence yet to justify routine endoscopy as a pivotal examination in halitosis assessment. On the other hand, a flexible laryngoscopy performed by an expert ENT specialist is most likely to bring contribution to halitosis assessment and further treatment.

Sometimes an internist referral is important when rare causes of halitosis are searched. Cases of rare diseases diagnosed trough halitosis complaint are refereed in literature. Dietary assessment, oral hygiene education and psychological advice are essential in halitosis treatment.

In our clinic, dentists, ENT specialists, gastroenterologists, nutritionists and psychologists work together. We use VSC interscan halimeter test as a routine in halitosis assessment. Each specialist perform specific tests concerning his/her own field only when they judge it is essential (i.e., ENT requests for laryngoscopy, gastroenterologist request for endoscopy). There are no unnecessary examinations and virtually all patients have a diagnostic after the assessment protocol. Following this methodology for the last seven years, we have a very high percentage (91%) of patients satisfied with the treatment^30^.

Finally, one must keep in mind that the patient suffering of halitosis is a person looking for help, often anxious and suspicious of any treatment, due to bad experiences using traditional approaches.

Halitosis must be treated as a serious condition, a multifactorial and a rational approach are essential for good results.

**Figure 3:**
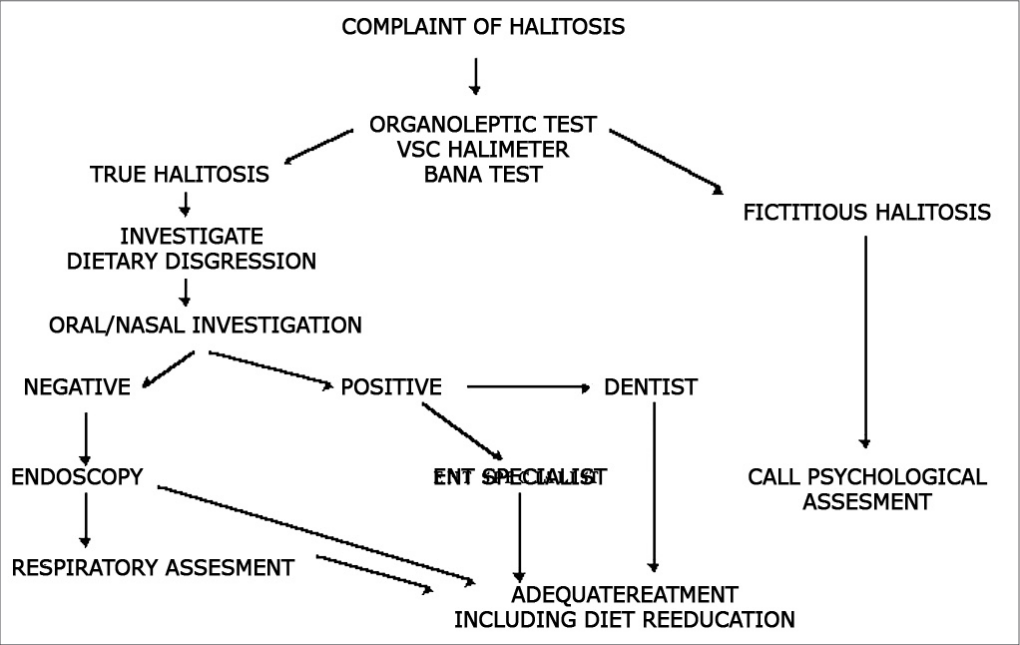
Flowchart suggested for halitosis assessment.

A flowchart for assessment of halitosis is suggested in Figure 3
